# Review of Articles and Discussions on Root Canal Treatment

**Published:** 1915-09

**Authors:** R. Ottolengui


### REVIEW OF ARTICLES AND DISCUSSIONS ON ROOT CANAL TREATMENT.

BY R. OTTOLENGUI, D.D.S.

During the winter we have been withholding various articles dealing with the subject of root canal treatment and filling, and the treatment of periapical infections, with the intention of publishing them all together as a symposium, that the student might have varying opinions in juxtaposition for his consideration. The material, however, proved too voluminous for the space available in a single issue, so that some appeared last month and more will be found in this number.

### Root Canal Pastes.

The use of a plastic substance of some sort, which in theory at least will fill a canal if all pulp tissue be removed, and preserve what is left, if any be left, maintaining the same in a condition immune against the attacks of micro-organisms, is so attractive a method that it is not strange that the recommendation, varying only in formula, is presented so frequently. If comment is now made upon Dr. W. I. Prime's communication, it is by no means personal, but merely to emphasize the errors of his proposition as a lesson to hundreds of others who hold quite the same notions and practice in quite the same way, only, perhaps, without the courage to confess in print.

The salient points in Dr. Prime's paper are: First, he never uses the dam, but trusts to washing out canals with tepid water and instrumentation to cleanse his canals. Then a paste is forced in until the patient gives a signal for stopping, because pain is felt. In the presence of putrescence he relies on phenol applied on paper cones and repeated till there is no odor.

Thus, in spite of all that has been written of the dangers of systemic infection from periapical abscesses, this practitioner is willing to recommend over his own signature the treatment of root canals unprotected from the bacteria in the oral cavity, and to count a root canal to be sterilized on the day when he can not detect the presence of bacteria with his olfactory organ. It is to be hoped that Dr. Prime and others who share his views will really read all the papers and discussions in relation to root canal work in this and the last issue, and that they may profit by what the real students have to say.

Reverting, however, to the subject of canal pastes, so long as men can purchase these sure-pop cures for all root canal troubles and rely upon the lies on the labels to salve their consciences for adopting these lazy man's methods, it is almost useless for the chemical scientists to declare that the pastes are worthless.

### Duty of the National Dental Association.

If the reorganized National Dental Association really means to emulate the methods of the American Medical Association, after which it has been patterned, it is high time for the Research Commission to attack this question of nostrums. All these canal pastes should be scientifically tested, and when proof has been accumulated that they are not only worthless, but actually harmful, since by the engaging advertisements that go with each package, dentists are tempted to fill canals with them, with the result that thousands of innocent patients have their health and lives endangered, then the National Dental Association should petition the Federal authorities to prohibit the further sale of such dangerous compounds.

We have been asked whether the dental profession will take the next step which must be taken in preventive medicine. This is the step that should be taken. The prevention of the continued poisoning of the people by abscesses resulting from the use of these baneful nostrums. We have been praying for this grand National Dental Association, and bragging of what we would do with the organization. Well, the day has arrived; we have the organization; the opportunity is ours. What are we going to do about it?

### Antiseptic Canal Operations.

Dr. C. Edmund Kells denies the possibility of aseptic root canal operations, and advocates the use of antiseptics; but in this symposium he will find none to agree with him. Not only is it declared that the aseptic root canal operation is possible, but both medical men and dentists claim that it is worse than useless to rely upon antiseptics. The classic surgery of today removes all infected tissue with aseptic instruments and hands, after which dressings are used, not charged with antiseptics as formerly, but with such agents only as will maintain the immunity of the wounded parts, contributing to healthy granulation tissue, and serving as a barrier against the inroads of pathogenic micro-organisms. This is just as essential in root work, or in periapical surgery, as elsewhere in the body, and no other course offers the slightest promise of a permanent cure.

It is true, as Dr. Kells says, that the vast majority of dentists today are not doing aseptic surgery when dealing with root canals; it is, as he announces, a pernicious practice to wrap cotton on a broach with dirty fingers and place that cotton as a dressing into a root canal. But it is possible for men to wrap cotton on their broaches with their fingers covered with rubber shields taken fresh from alcohol jars and using cotton which is continuously kept in the sterilizer.

No, it will not answer to rely on antiseptics, because our fingers are dirty. We must recognize the danger to our patients of treating their root canals in any but the most aseptic fashion, and then we must sit at the feet and learn from those who have perfected aseptic methods of practicing in this important field.

### Protrusion of Canal Fillings Through Foramina.

Dr. Clarence Grieves in this issue, in discussing Dr. Best's paper warns against the protrusion of a canal filling through the end of a root, while Dr. Rhein and others contend that no root canal which has been putrescent can be counted as properly filled unless the canal filling passes quite through the foramen, thus perfectly sealing the same.

Thus is set up a distinct difference of opinion, and the sooner this controversy is determined, the better, because of the prominence of the two men. Certainly Dr. Rhein's long and arduous labor in this field, and the fact that he has practiced his method for a great number of years, keeping voluminous radiographic records and case histories, entitles his views to the most serious consideration. On the other hand, the fact that Dr. Grieves cites Drs. Black and Noyes to the defense of his views in regard to renewed growths of cementum about the ends of the teeth, makes one feel that the subject must again be carefully studied.

Dr. Grieves tells us that these growths are physiologic, while Dr. Rhein declares that they are pathologic. Dr. Grieves describes what he calls secondary cementum as "a process of normal cementum formation which continues throughout life," etc., etc. But he does not make it clear that this can occur, or that it does occur after the death or removal of the pulp; and if it does, why should it be counted physiological? Is secondary dentine, the product of a perverted stimulus set up within the pulp by some irritating agency, a physiological or a pathological tissue?

Dr. Grieves opposes even the encapsulation of the end of a diseased root with gutta-percha, and declares, even where regeneration of bone is shown by radiographs under such conditions, that he can detect an area of rarefaction close to the gutta-percha and just at the apical foramen. Of this he says: "There is always some point where bone is just about to be formed, but is not."

This argument is based largely upon a reading, or perhaps upon a misreading of the radiographs which have been exhibited. The places which Dr. Grieves claims show rarefaction are merely places in which no actual bone has been laid down, or else where the bone, if present, is not sufficiently dense to stop the passage of the X-ray. This is not rarefaction in the sense in which the term is used in diagnosing diseased tissue, nor to the eye of an expert does one look at all like the other.

It would, of course, be a satisfaction to those that claim they can cure periapical disease to be able to prove conclusively the entire filling in of the abscess cavity with new bone. But the success of their advocated methods and the soundness of their doctrines do not depend upon the restoration of new bone. It is quite sufficient if the parts are restored to health and all disease eradicated. These men would be quite contented if skillful dissection, by competent anatomists, would find those little places near the foramina which Dr. Grieves calls rarefied, to be filled with healthy granulation tissue. That this is the case is most probable. That these so-called rarefied areas are diseased, or even prone to disease, case records abundantly prove not to be the case.

During the discussion Dr. Grieves challenged those present to tell him how to differentiate between the curable and incurable cases. None replied, but there is no difficulty about replying. Those who are skilled in reading radiographs can readily detect a truly necrotic apex, and in such conditions, apicoectomy usually is a reliable procedure, though, as Dr. Grieves truly points out, not so useful in the molar region. Where there is no actual death of the end of the root, or where its existence can not be positively determined, aseptic root canal treatment and filling promises a cure, and if the promise be not fulfilled the end of the root may even then be amputated, with the assurance that the main portion of the root is not a contributing agent in the continuance of the disease.

Of course, Dr. Grieves argues soundly when he declares that with the patient ill in bed, suffering from infection which might be traced back to the tooth, the internist is entirely within his rights when he sacrifices a tooth or teeth rather than risk the life of his patient.

But Dr. Rhein also is correct in making a plea for the salvation of all teeth where opportunity affords. If the patient is not bed-ridden, and can make the visits to the office, it not infrequently happens that from the very initiation of treatment a betterment of conditions begins, so that the patient is increasingly able to endure the sittings needed. And in the end he not only has his health, but likewise his tooth or teeth.

It is to be hoped that Dr. Grieves will continue his researches, as well as all the others who are at present teaching and preaching better methods, with the hope of attaining better results.

### Callahan's Methods.

Prominent in this field we have always had Dr. Callahan, and his two papers in this issue should be carefully studied. We have long had his views on the use of sulfuric acid, but it is most interesting now to read what he says of the use of sulfuric acid after the utilization of sodium-potassium. And one is immediately desirous of experimenting with the process suggested by Dr. Herman Prinz; the substitution of hydrochloric acid and sodium dioxide for the sulfuric acid and bicarbonate of soda. If the results are but half so good as promised they will be most useful.

In regard to Dr. Callahan's rosin method, that likewise seems a very rational and useful proposition. But when he speaks of pumping a cone up and down in a canal forty or fifty times, finally getting it to the end and forcing the rosin into the dentinal tissues, it is evident that he can enlarge canals to a larger bore than most men.

### The Problem Yet to be Solved.

It would seem, then, that we have practically perfected the technic of chemically cleansing out canals, and where they are large enough we have most satisfactory methods of filling them. We still need a safe mode of enlarging the canals after cleansing, so that we neither fill them up again with debris nor drill false pockets into or through the sides of the canals.

For this we require a better system of instrumentation, and for this we must have better instruments than have yet been devised. But several minds are at work on this problem, and it will undoubtedly be solved.—Editorial in August *Items of Interest.*

